# Pre-Treatment Amygdala Volume Predicts Electroconvulsive Therapy Response

**DOI:** 10.3389/fpsyt.2014.00169

**Published:** 2014-11-26

**Authors:** Freek ten Doesschate, Philip van Eijndhoven, Indira Tendolkar, Guido A. van Wingen, Jeroen A. van Waarde

**Affiliations:** ^1^Department of Psychiatry, Rijnstate Hospital, Arnhem, Netherlands; ^2^Department of Psychiatry, Radboud University Medical Center Nijmegen, Nijmegen, Netherlands; ^3^Donders Institute for Brain Cognition and Behavior, Centre for Neuroscience, Nijmegen, Netherlands; ^4^Faculty of Medicine, LVR Clinic for Psychiatry and Psychotherapy, University of Duisburg-Essen, Essen, Germany; ^5^Department of Psychiatry, Academic Medical Center, Amsterdam, Netherlands; ^6^Brain Imaging Center, Academic Medical Center, Amsterdam, Netherlands

**Keywords:** electroconvulsive therapy, response, amygdala, predictive value, structural MRI

## Abstract

**Background:** Electroconvulsive therapy (ECT) is an effective treatment for patients with severe depression. Knowledge on factors predicting therapeutic response may help to identify patients who will benefit most from the intervention. Based on the neuroplasticity hypothesis, volumes of the amygdala and hippocampus are possible candidates for predicting treatment outcome. Therefore, this prospective cohort study examines the predictive value of amygdala and hippocampal volumes for the effectiveness of ECT.

**Methods:** Prior to ECT, 53 severely unipolar depressed patients [mean age 57 ± 14 years; 40% (*n* = 21) male] received structural magnetic resonance imaging (MRI) at 1.5 T. Normalized amygdala and hippocampal volumes were calculated based on automatic segmentation by FreeSurfer (FS). Regression analyses were used to test if the normalized volumes could predict the response to a course of ECT based on the Montgomery–Åsberg Depression Rating Scale (MADRS) scores.

**Results:** A larger amygdala volume independently and significantly predicted a lower post-ECT MADRS score (β = −0.347, *P* = 0.013). The left amygdala volume had greater predictive value for treatment outcome relative to the right amygdala volume. Hippocampal volume had no independent predictive value.

**Conclusion:** A larger pre-treatment amygdala volume predicted more effective ECT, independent of other known predictors. Almost all patients continued their medication during the study, which might have influenced the course of treatment in ways that were not taken into account.

## Introduction

Electroconvulsive therapy (ECT) is an effective treatment for severely depressed patients (1, 2). ECT is a safe treatment option, however, it is regarded as invasive and some cognitive adverse effects are known (3). Although ECT is widely used, factors predicting ECT outcome are largely unknown. Knowledge on such factors might help to identify patients most likely to benefit from the intervention, and allow patients and clinicians to make better-informed decisions about initiating ECT. Several clinical and treatment characteristics were associated with increased response rates to ECT, including the presence of psychotic symptoms (4, 5), previous response to ECT (6), higher administered electrical stimulus intensity (7), bilateral electrode positioning (8), and higher age (9).

Structural neuroimaging could potentially be used to determine brain characteristics that predict ECT response, but until now structural MRI characteristics were not well established and showed contradictory results. For example, larger right hippocampal volumes predicted a poorer outcome after a course of ECT (10), whereas another study using visual rating scales to score severity of atrophy, showed that atrophy in the medial temporal lobe (MTL; a part of the brain including hippocampus and amygdala) seemed to be negatively correlated with the response to ECT (11).

The structural volumes of specific brain areas are partly determined by regional neuroplasticity. According to the neuroplasticity hypothesis, knowledge on regional plasticity of specific brain areas gives further insight into the pathophysiology of depression and its treatment. Among other factors, regional neuroplasticity was associated with the level of brain-derived neurotrophic factor (BDNF; i.e., a factor involved in the regulation of neuronal growth) (12). Contradicting results show that, within the MTL, both increased and decreased regional brain volumes as well as BDNF levels were associated with severe depression and its treatment by ECT (13, 14).

Firstly, bidirectional alterations in plasticity of the amygdala, a brain region that bears an important function in emotional memory and fear conditioning (13), were suggested to be related to depression. Also, patients suffering from depression showed both larger and smaller amygdala volumes relative to healthy controls [for a review, see Ref. (15)]. Secondly, depression was associated with relatively small volumes of the hippocampus (16). Moreover, a study using animal models of depression showed that BDNF knock-down in specific sites of the hippocampus resulted in depression-like behavior (17). The hippocampus is related to the declarative memory system and seems to mediate the more cognitive aspects of depression (e.g., feelings of worthlessness and guilt) (18).

Emphasizing the involvement of plasticity in the treatment of depression, studies on ECT and electroconvulsive shock (ECS; i.e., an animal model of ECT) showed that repeated ECT/ECS partially reverses the relatively high and low BDNF levels found in the amygdala and hippocampus, respectively, of depressed patients (19–24). Also, a longitudinal pilot study showed an increase of both amygdala and hippocampal volume due to an ECT course (25).

Recapitulating, according to the neuroplasticity hypothesis of depression, the hippocampus, and the amygdala seemed to be important structures in depression and the treatment with ECT. This study investigates whether individual differences in pre-treatment volumes of the amygdala and hippocampus predict the level of depression after a course of ECT.

## Materials and Methods

### Patients

**Figure 1:**
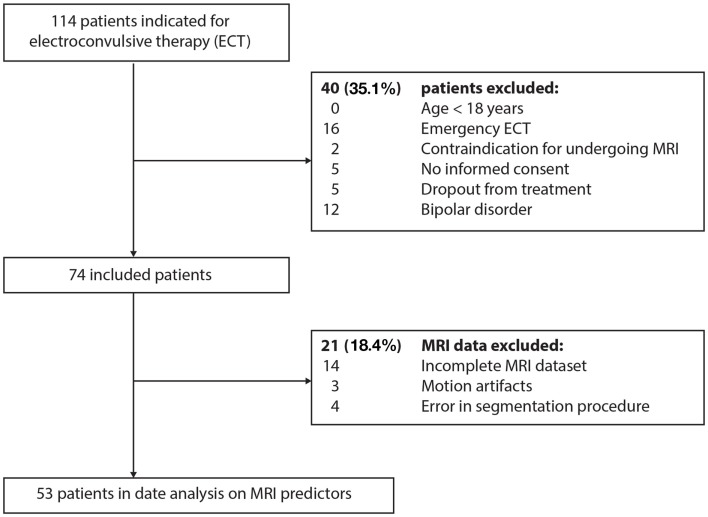
**Flow chart of the patient selection process**.

Only severely unipolar depressed patients classified according to the Diagnostic and Statistical Manual of Mental Disorders, 4th edition, Text Revision (DSM-IV-TR), indicated for ECT at the Rijnstate Hospital (Arnhem, the Netherlands), were selected for this study. Patients were excluded if aged <18 years, if they suffered from bipolar disorder, if there were contraindications for MRI of the brain, and if they dropped out of treatment during the ECT course (Figure 1).

The Medical Ethical committee of the hospital approved the research protocol of this study (NL24697.091.09). Characteristics of the patients are described in more detail elsewhere (26). After receiving full information about the study, written informed consent was obtained from all participants.

### Instruments

#### Psychometric instruments

Information on age, sex, psychiatric diagnosis, and previous ECT treatments were derived from the medical records. The severity of depression was determined with the MADRS by a trained research nurse. The MADRS consists of questions in 10 subcategories (27), rated 0–6, resulting in a score ranging from 0 to 60 (highest level of severity). Remission was defined as a post-ECT MADRS score of ≤10 points (28). Clinical raters were blind to the MRI results.

#### MRI and volumetric analysis

Imaging was performed within 1 week before the first ECT session on a 1.5 T MRI scanner (Philips Medical Instruments, Best, the Netherlands), using an eight-channel SENSE head coil. The scanning protocol included a high resolution T_1_-weighted (T_1_W) turbo field echo MRI (sequence parameters: repetition time = 7.6 ms; echo time = 3.5 ms; flip angle = 15°; 145 sagittal slices; voxel size = 1.1 mm isotropic). Prior to analysis, all raw MRI data were visually checked for the presence of (motion) artifacts. Regional volumes were established using the FS automatic subcortical segmentation tool, in which each voxel in the normalized brain volume is assigned one of about 40 labels, including hippocampus and amygdala^1^. Briefly, the processing stream comprises removal of the skull and dura, automated Talaraich transformation, defining the gray–white matter boundary, intensity normalization, and segmentation of subcortical gray matter. We manually corrected the cases where dura was included in the gray matter. The FS application enables automatic labeling of subcortical structures using a probabilistic algorithm. Initially, each image is a rigid body registered to a probabilistic atlas based on manually labeled image. Thereafter, the image is morphed to the atlas by a non-linear transform and a Bayesian segmentation procedure is employed. Each voxel in the MRI volume is automatically assigned to a neuro-anatomical label based on probabilistic information estimated from a manually labeled training set. The labeling procedure is not biased by anatomical variability. The segmentation procedure is based on three types of probabilities to disambiguate labels: first, the likelihood that a given structure occurs at a specific atlas location. Second, the likelihood of the image intensity given the tissue class. Third, the probability that a voxel belongs to a given tissue class based on likelihood of the spatial configuration of labels^2^. For each patient, the total volumes of the amygdala and the hippocampus were normalized by dividing each established volume by the total intracranial volume.

#### Treatment course and procedure

Electroconvulsive therapy was administered using a constant-current (0.9 A), brief-pulse [0.25 ms in right unilateral ECT (RUL) and 0.5 ms in bifrontotemporal (BL) ECT] device (maximum output 1008 mC; Thymatron IV; Somatics Incorporation, Lake Bluff, IL, USA), after induction of anesthesia intravenously with etomidate (0.3 mg/kg body mass), muscle paralysis with succinylcholine (0.5–1 mg/kg body mass) intravenously, and with appropriate oxygenation (100% oxygen, positive pressure) until the resumption of spontaneous respiration. Electrode placement was started RUL, except in patients at high risk for suicidality and/or acute somatic complications of the depression, or if previous BL ECT had successfully been administered. Dosage was set at 6-times initial seizure threshold (IST) in RUL ECT and at 2.5-times IST for BL treatment. Patients were treated twice weekly. RUL electrode placement could be changed into BL electrode placement during the ECT course if the patient did not show (enough) improvement after six RUL sessions, based on the clinical decision of experienced psychiatrists. One week before starting ECT, baseline MADRS scores were determined. The course of ECT treatment was terminated when mood had not further improved in the last two ECT sessions, based on the clinical decision of the psychiatrists. Within 1 week after the last ECT session, the post-ECT MADRS score was established.

### Statistical analysis

Data are presented as means ± SD or numbers or percentages when appropriate. The statistical assumptions for the regression analyses were tested in advance. For the assumption of linearity, residuals versus predicted values plot was created and interpreted. The Durbin–Watson and the Shapiro–Wilk test were used to check for violations of independence and normality, respectively.

Regression analyses were used to test if the normalized volumes of hippocampus and amygdala predicted the effectiveness of ECT. Primarily, the post-ECT MADRS score was the dependent variable in regression analyses of covariance. The independent variables were the normalized volumes of the amygdala and hippocampus, adjusted for age, sex, baseline MADRS score, presence of psychotic symptoms, and previous ECT treatment. These last two variables were added because of their known predictive value for ECT effectiveness (26). To assess whether post-ECT MADRS scores could also be predicted for out-of-sample cases, we used cross-validation. We computed the correlation between leave-one-out predicted scores and the observed scores, and tested for a positive association using permutation testing with 1000 randomizations in Matlab (R2014a).

For further confirmation, binary logistic regressions were used to test if the normalized volumes were predictive of the remitted group (post-ECT MADRS score ≤ 10) relative to the non-remitters. In this analysis, remission or not served as the dependent variable, and the normalized volumes as independent variables, adjusted for age, sex, baseline MADRS score, presence of psychotic depression and previous ECT treatment. To examine whether the effect was lateralized, analyses that produced significant effects were repeated separately with the left and right area volumes as independent variables. Additionally, since both BL and RUL electrode placements were applied, the eventual electrode placement (i.e., the electrode placement that was used to complete the treatment) was included as a possible mediator in an analysis of covariance (ANCOVA) in which the same variables were included as in the regression analysis. In all tests, *P* < 0.05 denoted statistical significance; SPSS for Windows (version 20) was used for the analyses.

## Results

### Patient characteristics

The study included 53 severely unipolar depressed patients, with a mean age of 57 ± 14 (SD) years, of which 21 (39.6%) were male. Out of 53 patients, 33 (62.3%) patients used benzodiazepines, 35 (66%) used anti-depressants, 34 (64.2%) used anti-psychotics, and 2 (3.8%) used anti-epileptics. Most patients (94.3%) suffered from longstanding, recurrent depressive disorder, and 16 (30.2%) patients were previously treated with ECT. The mean baseline MADRS score was 36.0 ± 8.1 (SD) points and psychotic symptoms were present in 12 (22.6%) patients (Table 1). The mean number of treatment sessions in a completed ECT course was 18.2 ± 7.4 (SD). At the initial session, 42 patients (79.2%) were treated with RUL ECT. Twenty (37.7%) of these patients switched from RUL to BL treatment, leaving 22 (41.5%) patients who were eventually treated with RUL treatment. After ECT, the mean MADRS score decreased significantly [*t*(52) = 13.07, *P* < 0.001] to a mean score of 13.5 ± 10 (SD). After the ECT course, 26 (49.1%) patients achieved complete remission. The patients who achieved remission did not significantly differ from the non-remitting patients in age (*P* = 0.213), sex (*P* = 0.958), total number of ECT sessions (*P* = 0.151), or use of concomitant medication [i.e., benzodiazepines (*P* = 0.227), anti-depressants (*P* = 0.920), anti-psychotics (*P* = 0.344), and anti-epileptics (*P* = 0.172)]. Also, comparison of the patients that were eventually treated with RUL and BL electrode placement revealed no differences in age (*P* = 0.748), sex (*P* = 0.474), total number of ECT sessions (*P* = 0.734), or use of concomitant medication [i.e., benzodiazepines (*P* = 0.464), anti-depressants (*P* = 0.786), anti-psychotics (*P* = 0.227), and anti-epileptics (*P* = 0.233)].

**Table 1:** **Patient and anatomical magnetic resonance imaging (MRI) descriptives at baseline of severely unipolar depressed patients (*n* = 53) treated with electroconvulsive therapy (ECT)**.

	Mean ± SD or *n* (%)
**Patient characteristics**
Age (in years)	57.1 ± 14.3
Male gender	21 (39.6)
MADRS score at baseline	36.0 ± 8.1
Presence of psychotic features	16 (30.2)
Previously treated with ECT	12 (22.6)
Total number of sessions	18.2 ± 7.4
**Concomitant medication**
Benzodiazepines	33 (62.3)
Anti-depressants	35 (66.0)
Anti-psychotics	34 (64.2)
Anti-epileptics	2 (3.8)
**MRI volumes of regions of interest (in mm^3^)**
Bilateral amygdala	4741 ± 624
Bilateral hippocampus	6711 ± 972

*SD, standard deviation; MADRS, Montgomery–Åsberg Depression Rating Scale*.

### ECT outcome related to the volume of amygdala and hippocampus

**Figure 2:**
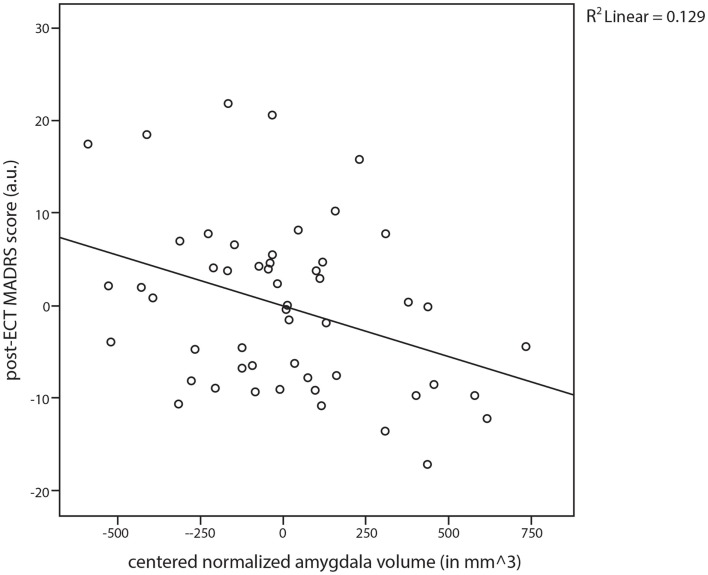
**Scatter plot and regression line for the post-electroconvulsive therapy (ECT) established Montgomery–Åsberg Depression Rating Scale (MADRS) scores against the normalized amygdala volume (amygdala volume/intracranial volume)**.

Adjusted for age, sex, baseline MADRS score, presence of psychotic symptoms, previous ECT course(s), and eventual electrode placement, the linear regression analyses of covariance revealed that the normalized bilateral amygdala volume independently predicted 12.9% of the variance in the post-ECT MADRS score within this specific model. Specifically, a larger pre-ECT normalized amygdala volume predicted a lower post-ECT MADRS score (Figure 2; β = −0.347, *P* = 0.013). Moreover, as expected, the presence of psychotic depression (β = −0.406, *P* = 0.014) and previous ECT treatment(s) (β = −0.344, *P* = 0.021) showed significant predictive values for lower post-ECT MADRS scores. In this model, the normalized bilateral hippocampus volume did not predict the post-ECT MADRS score (β = −0.065, *P* = 0.691).

To assess whether post-ECT MADRS scores could also be predicted for new cases on the basis of bilateral amygdala volume after correction for covariates, we calculated predicted post-ECT MADRS scores using linear regression with leave-one-out cross-validation. The correlation between predicted scores and observed scores approached significance (*r* = 0.274, *P* = 0.068), suggesting that ECT outcome can be predicted by a combination of amygdala volume and clinical variables.

Adjusted for age, sex, baseline MADRS score, presence of psychotic symptoms, previous ECT course(s), and eventual electrode placement, the binary logistic analyses confirmed our results by showing that a larger normalized amygdala volume also predicted remission after ECT (*P* = 0.028).

Identical regression analyses of the normalized left and right amygdala separately revealed a greater effect for the normalized left amygdala (β = −0.346, *P* = 0.013) compared to the normalized right amygdala (β = −0.245, *P* = 0.080).

## Discussion

This prospective cohort study in severely depressed patients showed that a larger pre-treatment amygdala volume predicted lower post-ECT depressive symptom score and remission after ECT. More specific, a larger left amygdala predicted better outcome of ECT. By contrast, the hippocampal volume had no predictive value for treatment outcome.

A study in elderly patients showed that more MTL atrophy correlated with less response to ECT (11). Because this study determined atrophy on a four-point rating scale by subjective rating of a radiologist, no differentiation between amygdala and hippocampal volume could be made. Our present study showed a correlation between ECT response and pre-ECT amygdala volume and not the hippocampus volume, rather than the MTL as a whole.

Remarkably, we could not replicate a correlation between poorer treatment response and larger pre-ECT hippocampal volume (10). Several factors might have affected this discrepancy in results. For instance, this might be due to the fact that Lekwauwa and colleagues did not adjust for any confounding factors. In our analyses, age (*r* = −0.463, *P* < 0.001), sex (*r* = −0.270, *P* = 0.025), and presence of psychotic features (*r* = −0.265, *P* = 0.028) all showed significant partial correlation with the normalized hippocampal volume. Therefore, one of these factors might have confounded the previously reported correlation between hippocampal volume and ECT response (10).

To assess whether ECT outcome could also be predicted for new cases, we predicted post-ECT depressive symptom scores for individual patients on the basis of data from the rest of the group using leave-one-out cross-validation. The result approached significance and tentatively suggests that the outcome of ECT can be predicted on the basis of a combination of amygdala volume and clinical variables. This result provides further support to our recent report that ECT outcome can be predicted using neuroimaging data (29). In that study, we analyzed functional and structural MRI data on a voxel-by-voxel basis using machine learning. However, whereas functional MRI data were predictive of ECT outcome, structural MRI data were not. Several methodological differences may have contributed to the differences in results between the current and previous study. First, we defined the amygdala on an anatomical basis and analyzed its volume in isolation in the present study, whereas we analyzed brain morphology across the entire brain on a voxel-by-voxel basis in the previous study. Second, the regression analysis accounted for the influence of clinical variables such as psychosis or previous ECT, whereas our machine learning analysis did not. Third, the required effect size to detect significant effects is different, as we had an *a priori* hypothesis for the amygdala in the current study, whereas we explored effects across the entire brain in the previous study. Regardless of these differences, both studies suggest that the outcome of ECT can be predicted using neuroimaging data, and future studies may investigate whether combining structural and functional MRI data could provide even better predictions of ECT outcome.

To explain the prediction of a better treatment response by a larger pre-ECT amygdala volume, we will focus on the hypothesized functional role of the amygdala in recovery from depression. A study in medication-naive depressed patients showed that treatment response was dependent on the amygdala retaining its plasticity during the course of illness (30). In our present study, although patients suffered mostly from chronic, recurrent depressions, retaining plasticity may still be important in achieving remission after a course of ECT. Moreover, compared to pre-ECT, post-ECT activity was shown to be increased in the mediotemporal lobe (31–33), and more specifically in the amygdala (34). At last, other studies showed relatively large mediotemporal lobe volumes (35) and high intra-amygdalar functional amino acids (36) specifically in patients that responded positively to ECT. Thus, previous studies have shown that retaining plasticity during the course of illness and large post-ECT volumes of the amygdala correlated with a positive ECT response. We hypothesize that the pre-ECT amygdala volume is related to these findings. That is, a larger pre-ECT amygdala volume might reflect relatively higher levels of retained intra-regional plasticity and it might also facilitate larger post-ECT amygdala volumes. Thus, a large pre-ECT amygdala volume might predict positive ECT response because a larger volume reflects greater capacity for the amygdala to fulfill its functional role in the recovery of depression by ECT, suggested by previous research (30, 35, 36). However, the data in the present study was not suited to test this hypothesis, since no post-ECT MRI data was collected.

The present study had some strengths and limitations that should be mentioned. First, the prospective nature of the study diminished the potential sources of bias and confounding factors that were associated with the retrospective design. Furthermore, compared with previous studies, the present sample size was relatively large. On the other hand, almost all patients continued their medication during the study, which might have influenced the course of treatment in ways that were not taken into account. At last, the number of treatment sessions per patient was high relative to other studies [see, for example, Ref. (37)]. This is probably best explained by the high degree of treatment resistance of the patients who are treated with ECT in the Netherlands.

## Conclusion

In conclusion, in this group of severely, mostly longstanding and recurrent, unipolar depressed patients, a larger amygdala volume predicted a more favorable ECT outcome. Further research, applying several MRI techniques, is needed to replicate these results and extend the findings to other groups of patients and treatments. Clinically, if replicated in other samples, pre-ECT amygdala volume might help clinicians and patients to better predict treatment response and make better-informed decisions about initiating ECT.

## Author Contributions

All authors were involved in analysis and interpretation of the data. Jeroen A. van Waarde was responsible for data acquisitions. Freek ten Doesschate drafted the work, and it was revised by all other authors. All authors approve this version for publishing and agree to be accountable for all aspects of the work.

## Conflict of Interest Statement

Indira Tendolkar received a speaker’s honorarium from Servier, the Netherlands. The authors have no other conflicts of interests to disclose.

## Footnotes

^1^http://surfer.nmr.mgh.harvard.edu/

^2^http://surfer.nmr.mgh.harvard.edu/fswiki
